# Vitamin D and Risk for Vitamin A Intoxication in an 18-Month-Old Boy

**DOI:** 10.1155/2016/1395718

**Published:** 2016-07-10

**Authors:** Valentina Talarico, Massimo Barreca, Rossella Galiano, Maria Concetta Galati, Giuseppe Raiola

**Affiliations:** ^1^Department of Pediatrics, “Pugliese-Ciaccio” Hospital, 88100 Catanzaro, Italy; ^2^Department of Neonatology, “Pugliese-Ciaccio” Hospital, 88100 Catanzaro, Italy; ^3^Department of Pediatric Oncology and Hematology, “Pugliese-Ciaccio” Hospital, 88100 Catanzaro, Italy

## Abstract

An 18-month-old boy presented with abdominal pain, vomiting, diarrhea, and poor appetite for 6 days. He had been given a multivitamin preparation once daily, containing 50.000 IU of vitamin D and 10.000 IU of vitamin A for a wide anterior fontanelle for about three months. He presented with hypercalcemia, low levels of parathyroid hormone (PTH), and very high serum 25-hydroxyvitamin D (25-OHD) levels. Renal ultrasound showed nephrocalcinosis. He did not have sign or symptom of vitamin A intoxication. Patient was successfully treated with intravenous hydration, furosemide, and prednisolone. With treatment, serum calcium returned rapidly to the normal range and serum 25-OHD levels were reduced progressively. In conclusion the diagnosis of vitamin D deficiency rickets without checking 25-OHD levels may cause redundant treatment that leads to vitamin D intoxication (VDI).

## 1. Introduction

Vitamin D intoxication (VDI) is a rare event [1] that usually occurs as a result of improper use of pharmaceutical preparations of vitamin D and can lead to life-threatening hypercalcemia [2]. Hypervitaminosis A has been observed in children and can lead to raised intracranial pressure and serious sequelae [3]. Recognizing vitamin intoxication can be difficult as the initial symptoms of toxicity are nonspecific and they depend on many factors (e.g., dose taken and comorbidities). Clinicians should remain aware of this entity and elicit history regarding use, brand, and dosing of over-the-counter supplements in order to make a timely diagnosis and initiate treatment [4].

## 2. Case Report

**Figure 1:**
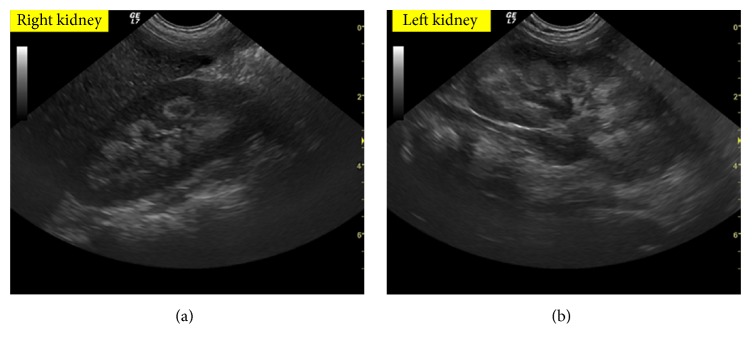
Examination of the right (a) and the left (b) kidneys: the kidneys are normal in size but exhibit multiple areas of increased echogenicity involving all of the medullary pyramids, without posterior acoustic shadowing; increasing beginning in the periphery of the renal pyramids.

An 18-month-old boy without previous health problems presented with abdominal pain, vomiting, and poor appetite for 6 days. The medical history revealed that he had been given a multivitamin preparation once daily (50.000 International Unit (IU) of vitamin D and 10.000 IU of vitamin A) by his pediatrician for a wide anterior fontanelle for about three months. The physical examination on admission revealed only agitation. Vital signs were normal. Serum calcium was 11.5 mg/dL (normal 8–10.4), phosphorus was 4.3 mg/dL (normal 4.5–5.5), alkaline phosphatase (ALP) was 91 IU (normal 60–321), creatinine was 0.5 mg/dL, 25-hydroxyl-vitamin D (25-OHD) levels were 2271 ng/mL (normal 30–100), and parathyroid hormone (PTH) was <3 pg/mL (normal 4.6–58.1). Urinary calcium/creatinine ratios were 1.1 mg/mg (normal <0.21). The patient was treated with intravenous hydratation of 150 mL/Kg/day and furosemide at 2 mg/kg/day, with a diet with low calcium and phosphorus content. Renal ultrasonography showed medullary nephrocalcinosis with symmetric bilateral involvement (Figure 1); so prednisolone 1 mg/kg/day was also added to the treatment regimen. The ECG, hearing test, and ophthalmological examination were all normal, in particular there was no “papilledema.” With this therapy, the total calcium level decreased rapidly and we observed a clear improvement in clinical conditions. When he was discharged, his 25-OHD level was still elevated at 630 ng/mL and his calcium concentration was 9.5 mg/dL. His discharge instructions included avoidance of products containing vitamin D. On follow-up examination at 1 month the patient was normocalcemic with normal urinary calcium excretion and 25-OHD levels were at 150 ng/mL. The patient is being followed up for nephrocalcinosis.

## 3. Discussion

Vitamin D intoxication (VDI) usually develops due to high dose of vitamin D given by health care providers before a clear diagnosis of vitamin D insufficiency or rickets is established [5]. Another cause of intoxication is the inappropriate administration of high dose of vitamin D in infants by families for complaints such as delayed teething, “late walking,” and “knock-kneed gait” [5]. According to the American Academy of Pediatrics, serum vitamin D levels above 100 ng/mL are considered as hypervitaminosis D, whereas levels above 150 ng/mL are associated with VDI [6]. There is no consensus on the dose of oral vitamin D that leads to intoxication; individual variability must be considered with VDI [6]. In 2011, the American Medical Institute estimated tolerable upper limits of vitamin D are 1000 IU/day for ages 0-1, 2500 IU/day for ages 1–3, 3000 IU/day for ages 3–8, and 4000 IU/day for age 9 and above [6]. So our patients, assuming 50.000 UI/daily, exceeded the level of toxicity of vitamin D by about 20 times. Children with VDI present with symptoms of hypercalcemia, such as poor appetite, weight loss, abdominal pain, vomiting, constipation, polyuria, and polydipsia, and in severe cases, life-threatening dehydration [1, 2]. Since vitamin D is lipophilic and stored in fat tissues, the effects of toxicity may last for months despite the removal of the exogenous source of vitamin D [6, 7].

In patients with VDI, hypercalcemia, normal or high serum phosphorus levels, normal or low levels of ALP, high levels of 25-OHD, low serum of PTH, and high urinary calcium/creatinine ratio are usually present [6]. Long-term hypercalciuria typically results in calcium storage in the epithelial basement membrane and tubular cells in the loop of Henle, as well as calcification at the corticomedullary junction. Medullary nephrocalcinosis can be detected on ultrasound better than in X-ray or computed tomography images. Nephrocalcinosis is a common finding in various pathological conditions characterized by hypercalciuria and/or hypercalcemia and only in 10% of cases it is associated with VDI. Treatment for VDI includes immediate removal of the exogenous source, intravenous fluid hydration, loop diuretics, low-calcium diet, and sometimes glucocorticoids [5]. The first line of therapy of hypercalcemia is intravenous hydration with normal saline to increase the glomerular filtration rate and calcium excretion [1, 6]. It can be combined with specific diuretics that increase calcium excretion, such as loop diuretics. Glucocorticoids and calcitonin can be added if symptomatic hypercalcemia persists despite hydration and diuretics. Glucocorticoids suppress the activity of calcitriol and reduce the production and activity of 1,25(OH)D2 and intestinal calcium absorption [1, 6]. Calcitonin inhibits bone resorption and blocks release of calcium and phosphonates into the serum [6]. Bone resorption is increased in VDI, and therefore, antiresorptive therapy with bisphosphonates, such as pamidronate and alendronate, can successfully lower serum calcium levels in children and adults [1]. They have been used for hypercalcemia of malignancy and metastatic bone disease in addition to osteogenesis imperfecta [7]. IV bisphosphonates have been proven to be effective in the treatment of VDI [6]. However, intravenous bisphosphonate therapy carry additional risk of chelation in vascular bed and may be associated with more severe side-effects [7]. In 2003, oral alendronate was used for the first time in an infant for treatment of hypercalcemia related to VDI [6, 7]. A few case reports appeared after this report demonstrating successful use of alendronate in VDI [7].

The patient described in this report was mistakenly given not only 50.000 IU/day of vitamin D, but also 10.000 IU/day of vitamin A, so there was also a high risk of vitamin A intoxication. Vitamin A toxicity presents with dry, scaly skin with areas of desquamation and fissuring of the lips. Other symptoms include headache, fatigue, anorexia, nausea, vomiting, blurred vision, pseudotumor cerebri, myalgias, and arthralgias [3]. Recommended daily intake of vitamin A is between 100 and 5000 IU [3]. Chronic toxicity results from the ingestion of high amounts of preformed vitamin A for months or years, but there is wide interindividual variability for the lowest intake required to elicit toxicity [8]. In children, however, hypervitaminosis develops quickly and usually resolves quickly [9].

In conclusion, this case led us to underline that, in accord with the international guideline, if we want to do therapy with vitamin D it is important before to assess blood levels of vitamin D and to avoid using higher dose of supplementation without monitoring.

Because of the risk of toxicity, a conservative approach to therapy for vitamin D deficiency in infants and young children should be considered [10]. On the other hand, parents of all infants should be asked whether they are using dietary or oral supplement, and serial questioning may be required during supplementation to avoid excessive intake [6]. Besides, we would like to stress that multivitamin preparations should not be used for vitamin D treatment, because there is possibility of a higher risk of multiple vitamin intoxication, and that patients unnecessarily treated with vitamin supplementation need to be evaluated for findings of hypervitaminosis [2].
